# Partial two-stage exchange: an alternative method for infected total hip arthroplasty

**DOI:** 10.1186/s12891-021-04550-9

**Published:** 2021-08-12

**Authors:** Mumingjiang Yishake, Lan Tang, Xi Chen, Yuejian Wang, Rongxin He

**Affiliations:** 1 Department of Orthopedic Surgery, The First Affiliated Hospital of Zhejiang Chinese Medical University, Zhejiang, Hangzhou China; 2 Department of Orthopedic Surgery, The Second Affiliated Hospital, School of Medicine, Zhejiang University, #88 Jiefang Road, Zhejiang, 310009 Hangzhou China; 3 Department of Public Health, Zhejiang University, Zhejiang, Hangzhou China

**Keywords:** Hip, Arthroplasty, Periprosthetic infection, Two-stage exchange

## Abstract

**Background:**

Total two-stage exchange is commonly used in clinical practice as a treatment for infected total hip arthroplasty (THA); however, this approach involves considerable limitations, including significant bone loss and severe trauma. This retrospective cohort study was conducted to evaluate clinical outcomes following the use of partial two-stage exchange (PTE) for infected THA.

**Methods:**

We performed a retrospective analysis of 28 patients with infected THA who were treated by PTE between September 2000 and June 2019. Eligibility for PTE was limited to patients with a well-fixed femoral stem prosthesis. In the first stage of the operation, the femoral stem prosthesis was preserved; subsequently, the acetabular prosthesis, liner, and head were replaced with an antibiotic-loaded spacer. The new prosthesis was then implanted into patients and monitored for at least 3 months to ensure freedom from infection.

**Results:**

Patients were followed for an average of 4 years (range, 2–11 years), with an overall success rate of 85.7% (24/28). The mean Harris hip score at the final follow-up was 76.2 ± 11.7 points.

**Conclusions:**

The findings of this study suggest that PTE could be an acceptable option for a subset of patients with infected THA, offering a satisfactory infection control rate and clinical outcomes comparable to those of total two-stage exchange, but with less harm.

## Background

Arthroplasty is currently recognized as the most effective method for treatment of advanced joint diseases [1]. Accordingly, the annual number of arthroplastic surgeries has increased rapidly in recent years, and is expected to exceed 4 million by 2030 [1]. Infection is a catastrophic complication of surgical operations, especially those involving arthroplastic procedures. The reported incidence rates of infection are 0.5–3.0% following primary total hip arthroplasty (THA) and 4.0–6.0% following revision THA, resulting in extremely high costs and poor clinical outcomes; these infections represent considerable burdens to both patients and society [2–4].

As the number of THA procedures increases, greater numbers of periprosthetic joint infections (PJIs) and revision surgeries are expected. Currently, total two-stage exchange (TTE) revision, which removes both the acetabular and femoral components and involves placement of an antibiotic-loaded spacer, is considered the standard of care for chronic PJI in the United States [2, 4, 5]. However, TTE remains a technically complex procedure requiring a long duration of hospitalization due to serious injury caused by the removal of all components [3–5]. Moreover, the removal of well-fixed femoral components using aggressive debridement inevitably sacrifices bone stock and compromises reconstruction fixation. Therefore, it is important to explore more effective surgical procedures.

In 2009, partial two-stage exchange (PTE) revision was proposed as an alternative treatment for PJI, as a method to address many of the limitations of TTE [6]. For patients with localized infections that do not involve well-fixed femoral components, PTE is able to reduce the level of surgical trauma, thereby accelerating the rehabilitation process [7]. However, a systematic search of PubMed, the Web of Science, and the Cochrane Library revealed only a handful of preliminary reports describing the use of PTE for infected THA, highlighting the slow adoption of this procedure in clinical practice [7–11]. In this retrospective study, we examined the use of PTE for infected THA, providing additional clinical evidence regarding the efficacy and reliability of this surgical procedure.

## Methods

### Patients

Between September 2000 and June 2019, a total of 35 patients with chronic PJI were treated by PTE at our institution. Of these, 2 patients were lost to follow-up and 5 patients were excluded from analysis due to insufficient follow-up (< 2 years). The remaining 28 patients (28 hips) were included in this retrospective study, consisting of 13 women and 15 men. The mean age at the time of surgery was 61 years (range, 40–78 years). The average body mass index was 23.7 kg/m^2^ (range, 18.4–31.7 kg/m^2^). The Harris hip score upon admission to the hospital was 39.8 ± 10.7 points. The underlying diagnoses for primary THA were osteoarthritis in 25 patients, posttraumatic arthritis in 1 patient, and femoral head necrosis in 2 patients. 21 primary THA procedures were performed at our institution; the remaining 7 primary THAs were performed at other hospitals. The baseline demographic data were listed in Table 1. Meanwhile, 135 patients with no significant difference in clinical conditions but receiving TTE treatment were included to compare the success rate of different treatment methods.

**Table 1:** Characteristics of patients who underwent PTE

Patient ID	Age (y)	Sex	Side	Diagnosis for primary THA	Organism identified	Duration of infection (m)	Interval Between surgeries (m)	Time of follow-up (y)
1	40	M	Right	OA	*S. epidermidis*	72	4	2.1
2	58	M	Right	OA	*S. aureus*	6	3	3.3
3	54	F	Right	OA	Culture-negative	24	3	3.7
4	69	F	Right	PA	*S. haemolyticus*	7	3	4.3
5	55	M	Right	FHN	CNS	12	6	4.0
6	65	M	Left	OA	CNS	16	5	3.6
7	60	M	Left	OA	*S. aureus*	24	12	11.3
8	55	M	Right	OA	Culture-negative	6	3	5.3
9	66	M	Right	OA	*S. aureus*	60	3	3.9
10	52	F	Left	OA	*S. haemolyticus*	6	4	2.4
11	47	F	Left	FHN	CNS	12	3	3.7
12	77	M	Right	OA	Culture-negative	12	3	3.6
13	73	F	Left	OA	Culture-negative	6	3	4.2
14	68	F	Left	OA	MRSA	7	6	3.3
15	50	M	Right	OA	*S. aureus*	24	3	3.7
16	64	F	Right	OA	Culture-negative	9	3	3.5
17	78	F	Left	OA	*E. coli*	12	3	7.8
18	58	F	Right	OA	Culture-negative	6	6	3.4
19	62	M	Right	OA	*S. haemolyticus*	8	6	3.3
20	66	F	Left	OA	*S. epidermidis*	60	3	3.5
21	61	M	Right	OA	*S. aureus*	6	3	3.5
22	74	M	Left	OA	MRSA	10	3	3.3
23	51	M	Left	OA	CNS	6	4	4.4
24	50	F	Left	OA	*S. epidermidis*	24	3	3.2
25	67	F	Left	OA	*S. haemolyticus*	7	6	3.3
26	47	M	Right	OA	CNS	6	4	5.1
27	68	F	Right	OA	*P. mirabilis*	12	3	3.2
28	72	M	Left	OA	*S. aureus*	6	3	2.2

*THA* total hip arthroplasty, *OA* osteoarthritis, *FHN* femoral head necrosis, *PA* posttraumatic arthritis, *CNS* coagulase-negative Staphylococcus, *MRSA* methicillin-resistant *staphylococcus aureus*

The diagnosis of deep infection was made based on the new definition for PJI established by the Musculoskeletal Infection Society workgroup [12]. Briefly, diagnosis of infection was defined as the presence of a discharging sinus communicating with the joint, growth of a microorganism from joint fluids or at least two separate tissues from the affected prosthetic joint, or by the presence of four of the following six criteria: (a) elevated C reactive protein (≥ 10 mg/L) and erythrocyte sedimentation rate (≥ 30 mm/h); (b) elevated synovial white blood cell count (≥ 2000/μL); (c) elevated synovial neutrophil percentage (≥ 65%); (d) presence of purulence in the affected joint; (e) isolation of a microorganism in the culture of periprosthetic tissue or fluid; (f) detection of > 5 neutrophils per high-power field on histopathologic examination [12]. The definition of chronic infection was based on the criteria of Tsukayama et al. [13].

The femoral components were preoperatively measured for loosening based on the criteria described by Harris [14] and Engh et al. [15]. Possible loosening of all femoral components was further confirmed during the operation. For well-fixed cementless stems where radiographic evidence indicated bone ingrowth along the entire length of the stem, the femoral stems were retained without any attempt to remove them [8, 15, 16], and a PTE revision was made. For patients with evidence of bone ingrowth in one-third or less of the proximal area and radiolucent lines around the distal portion of the stem on preoperative radiographs, a PTE revision was made if removal of the femoral stem failed [8, 15, 16].

### Surgical technique

All surgeries were performed by the same experienced, fellowship-trained arthroplasty surgeon. The basic principles of PTE revision were as follows: radical debridement, removal of the acetabular component and artificial femoral head, retention of the uninvolved femoral stem component, and insertion of an antibiotic-loaded cement spacer, followed by two-stage reimplantation.

**Fig. 1:**
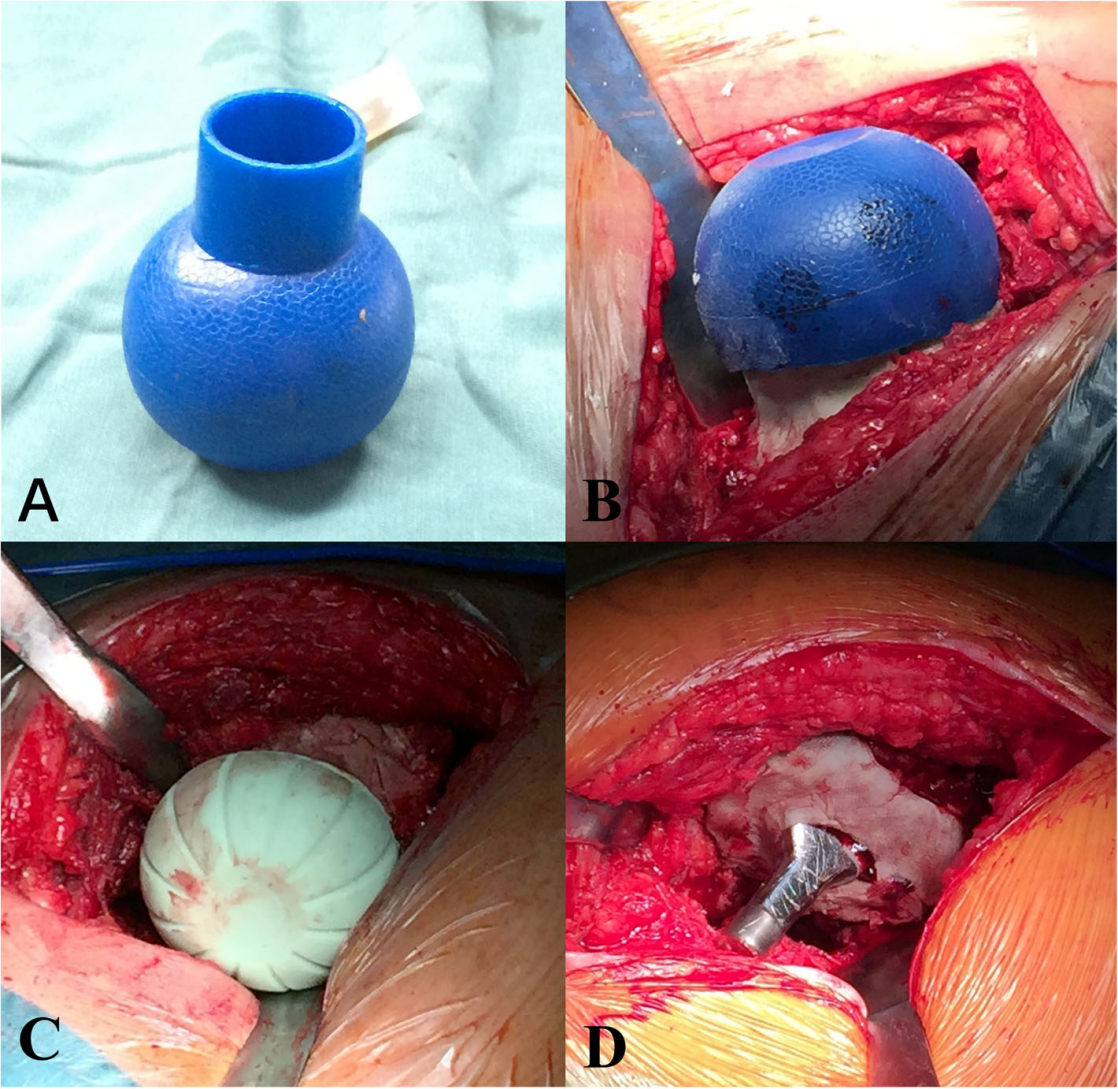
Partial steps of PTE treatment. (**A**) Select the bulb-shaped irrigation syringe (CR Bard, Inc) as a mold. (**B**) Cut the bulb-shaped irrigation syringe to the appropriate size and make the cement spacer. (**C**) The finished antibiotic-laden cement spacer. (D) we applied a small amount of antibiotic-loaded cement around the proximal end of femoral stem prosthesis, trying to reduce bone loss and block the bacteria from affecting the femoral stem components

In the first stage of the operation, all patients received radical debridement under general anesthesia through the original THA incision, which was extended distally when necessary. The acetabular component and artificial femoral head were then removed; the femoral stem component was retained in place. For each patient, at least two separate tissues from the affected prosthetic joint were harvested for intraoperative frozen biopsy and bacterial culture. Afterwards, the wound was successively flushed with 0.9% NaCl solution and hydrogen peroxide solution, followed by pulsatile lavage for 15 min in iodine solution. The removed components were then replaced with antibiotic-loaded cement spacers. These spacers consisted of high-viscosity cement (Palacos, Zimmer, Warsaw, IN, USA) and vancomycin [17]. The dose of vancomycin was determined based on previous studies (4 g vancomycin per 40 mg bag cement) [18, 19]. Spacers were handmade using a pediatric ear and ulcer syringe (outer diameter, 44 mm; CR Bard, Inc., Covington, GA, USA) or other suitable spherical tools when necessary. Additional antibiotic-loaded cement was applied around the proximal end of the femoral stem prosthesis to reduce bone loss and prevent bacteria from affecting the femoral stem components (shown in Fig. 1). At this stage, the acetabular bone defect would not be treated, and the patient would be told to avoid stress on the surgical side.

Postoperatively, a 6-week period of organism-sensitive intravenous antibiotic therapy was applied; all patients were observed for at least 3 months (mean, 4.7 months; range, 3–12 months). Serum C reactive protein level, erythrocyte sedimentation rate, and white blood cell count were monitored monthly. During this period, all patients were permitted to bear weight on the affected joint as tolerated.

Second-stage reimplantation was performed based on a combination of the patient’s general condition, wound healing without signs of infection (determined using the same criteria as initial diagnosis), and normalization of laboratory data. During the operation, the spacers were removed under general anesthesia through the original incision. Intraoperative pathological examination of frozen biopsy confirmed that the level of neutrophil granulocytes was < 5 per high-power field. Routine flushing of the wound was performed using 0.9% NaCl solution and hydrogen peroxide solution, followed by pulsatile lavage for 15 min in iodine solution. Patients with acetabular bone defects were treated following the common recommendation [20]. Generally, Paprosky Type I, Type IIA and Type IIB defects were managed with a noncemented, porous-coated hemispheric implant with or without the use of adjunctive screw fixation. Bone grafting was usually not considered. Paprosky Type IIC defects were treated with a noncemented, porous-coated hemispheric implant with the use of adjunctive screw fixation, and bone grafts were almost all required. The treatment of Paprosky type III defects often required supplemental porous metal augments such as tantalum metal blocks, as well as ‘cup-on-cup’ or ‘cup-cage’ technologies to reconstruct the acetabulum [21, 22]. Finally, the new prosthesis was implanted.

### Data recording and follow-up

Patients were followed up regularly in our clinic postoperatively at 1, 3, and 6 months, then once yearly thereafter. Perioperative parameters, wound condition, and Harris hip scores were evaluated to assess clinical outcomes. In this study, we defined failure as recurrence of infection in the same hip, requirement of additional surgical procedures for infection control, or use of long-term (> 6 months) suppressive antibiotics.

### Statistical analysis

Statistical analyses were conducted using SPSS Statistics, version 22.0 (SPSS, Chicago, IL, USA). Mean values and standard deviations were calculated for continuous variables; comparisons between groups were performed using a paired sample t-test. Pearson chi-squared tests were used to compare categorical data. The generalized estimating equations (GEE) and repeated measures ANOVA were used to assess the correlation between sorts of parameters that received multiple measures. *p* values < 0.05 were considered statistically significant.

## Results

**Fig. 2:**
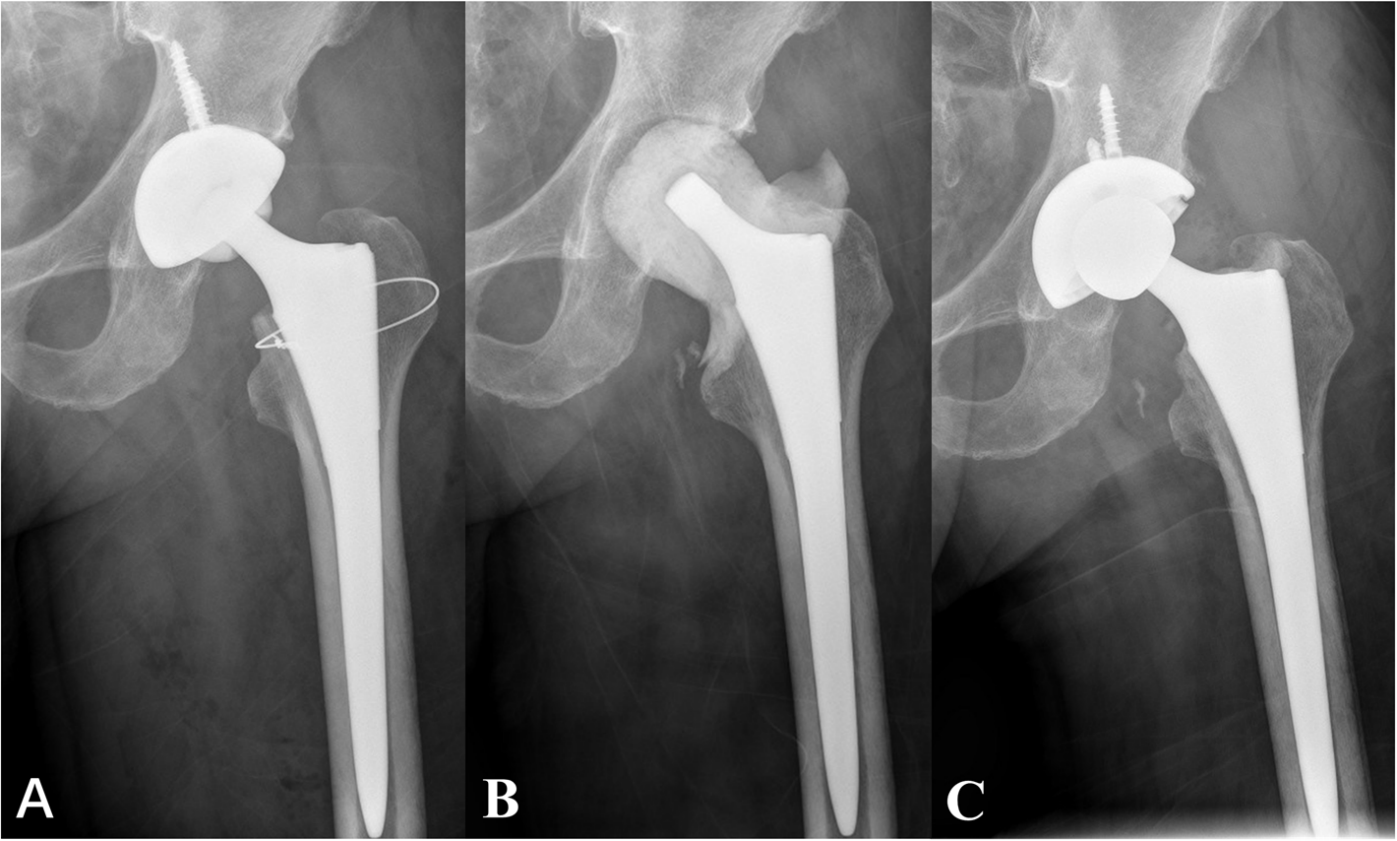
Left hip anteroposterior radiograph of patient 22. This patient underwent left THA 6 years previously. (**A**) The X-ray showed evidence of loosening of prosthesis but the femoral stem. (**B**) The acetabular cup was removed, and a cement spacer was inserted in the first-stage operation. (**C**) Until the 3-year follow-up, the patient was free of infection

**Fig. 3:**
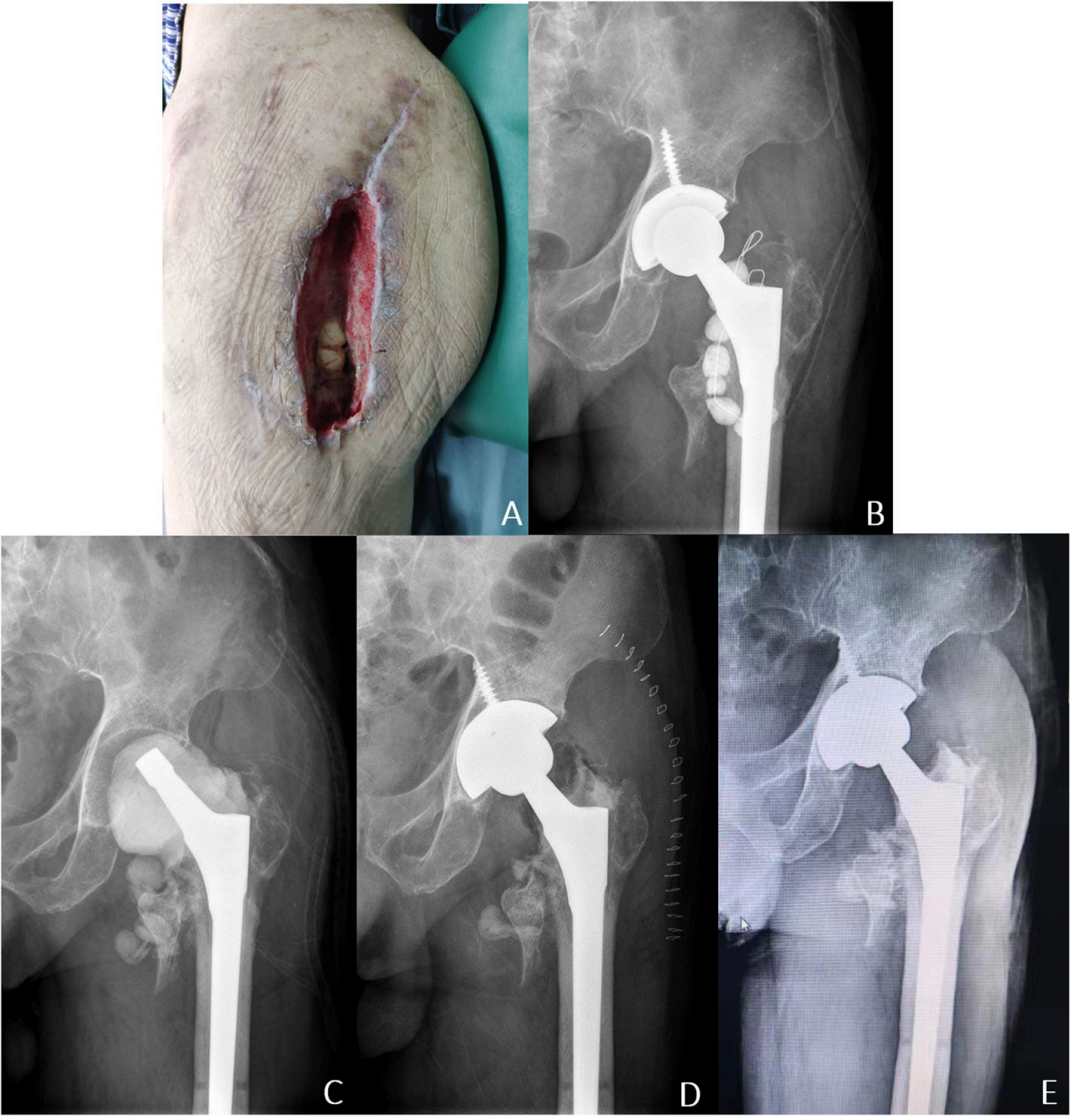
Left hip anteroposterior radiograph of patient 26. This patient who had undergone left THA half year previously was unfortunately infected. He underwent debridement and antibiotic-loaded cement implantation at the local hospital. (**A**) Photo taken when admission showed noticeable infection and massive soft tissue defects. (**B**) The X-ray showed no evidence of loosening of the acetabular cup and the femoral stem. (**C**) The acetabular cup was removed and a cement spacer was inserted in the first-stage operation. (**D**) At the second-stage operation, the spacer was removed, and the new implants were inserted. (**E**) There was no radiographic sign of implant loosening during the follow-up

We retrospectively analyzed 28 patients with chronic PJI who were treated by PTE at our institution. Patients were followed up for an average of 4 years (range, 2–11 years). The interval from the appearance of infection to the first surgery was 17 months (range, 6–72 months); the second-stage surgery was accomplished after a mean interval of 4.1 months (range, 3–12 months). The success rate of treatment in this study was 85.7% (24/28). Except for recurrent infection, no complications were observed in this study, such as deep vein thrombosis, implant loosening, nerve injury, dislocation, or death (shown in Figs. 2, 3).

At the final follow-up, inflammatory indicators such as erythrocyte sedimentation rate, C reactive protein level, and white blood cell count were within the normal ranges. Slight increases were evident in a handful of patients, although it is uncertain whether these changes were due to recurrent infection or complications from other unrelated diseases. The ESR of men declined more slowly than women after surgery in an age-dependent manner. The GEE results showed that there was a positive correlation between postoperative pain score and CRP values. The average Harris hip score at 1 month postoperatively was 51.8 ± 8.9 points, which increased to 61.3 ± 8.2 points at 3 months postoperatively and 76.2 ± 11.7 points at the final follow-up, revealing significant improvement in hip function (Table 2).

**Table 2:** Main indicators on patients

	ESR (mm/h)		CRP (mg/L)	Pain score	Harris score
	Male	Female			
Preoperative	68.9 ± 19.6	51.1 ± 25.0	73.8 ± 33.1	20.1 ± 6.9	39.8 ± 10.7
1st month	34.9 ± 18.7	29.0 ± 19.6	16.1 ± 9.6	24.2 ± 2.9	51.8 ± 8.9
3rd month	11.3 ± 5.6	14.4 ± 9.5	8.2 ± 3.9	35.0 ± 3.6	61.3 ± 8.2
Last follow-up	7.2 ± 2.7	6.7 ± 1.6	3.5 ± 2.6	42.1 ± 1.5	76.2 ± 11.7

*ESR* erythrocyte sedimentation rate, *CRP* C-reactive protein

A total of four treatment failures were observed in this study. Three were due to persistent infection before second-stage revision, while the fourth was attributed to recurrent infection after two-stage revision (Table 3).

**Table 3:** Information on patients who were defined as failures

Patient ID	Age (y)	Sex	Prior surgery	Comorbidities	Bacterial culture	Subsequent surgery	Follow-up time (y)
4	69	F	Revision THA	Skin defect	Negative	TTE	4.3
7	60	M	Primary THA	Smoker, hypertension	Negative	PTE after three debridements	11.3
17	78	F	Primary THA	Hypertension, diabetes, Cardiovascular infarction history	Negative	First-stage exchange only	7.8
26	47	M	Primary THA	Smoker	MRSA	TTE	5.1

*THA* total hip arthroplasty, *TTE* total two-stage exchange, *PTE* partial two-stage exchange, *MRSA* methicillin-resistant *staphylococcus aureus*

Failure 1: A 69-year-old woman had undergone prior hip operations due to trauma; she presented with poor local soft tissue condition, including severe scarring. This patient exhibited delayed wound healing, which manifested as redness and swelling. The patient’s serum erythrocyte sedimentation rate and C reactive protein level were abnormally high. After receiving radical debridement, she underwent TTE revision, resulting in successful control of the infection.

Failure 2: A 60-year-old man failed treatment due to a refractory infection that was detected after the first stage of revision. Three complete radical debridement surgeries were performed to control the infection, after which the patient underwent second-stage revision. Recurrent infection was not observed at the final follow-up.

Failure 3: A 78-year-old woman failed to complete the second-stage revision after careful evaluation due to advanced age and extremely poor health, as well as several underlying diseases (i.e., hypertension, mellitus diabetes, and prior cardiovascular infarction). She is currently using a wheelchair and has not experienced recurrent infection.

Failure 4: A 47-year-old man experienced recurrent infection at 6 months after second-stage revision. The results of bacterial culture were similar to those obtained prior to surgery. After complete debridement, TTE revision was performed. Recurrent infection was not observed at the final follow-up.

## Discussion

Compared with TTE, PTE is only indicated for a narrow subset of patients with PJI; however, no consensus has been reached regarding selection criteria for this procedure (Table 4) [6, 7, 9–11, 23–26]. Based on the data presented here, as well as the data from previous studies, we propose that the stricter eligibility criteria should be used for PTE revision for hip PJI [11]. We suggest the following inclusion criteria for PTE revision for chronic hip PJI: 1) chronic infection (> 6 months) after primary THA; 2) localized mild infection not involving the femoral component (assessed carefully based on laboratory and histopathological tests, both preoperatively and intraoperatively); 3) no sign of femoral component loosening, determined using the criteria described by Harris [14] for cemented femoral components and the system developed by Engh et al. [15] for cementless femoral stems; 4) fair condition of surrounding soft tissue without a history of multiple hip surgeries; and 5) Removal of the femoral component would lead to significant femoral bone loss and compromise of future fixation, or Elderly patients in worse health status with high risk of operation;

**Table 4:** Summary of evidence of PTEs

Reports	Year	Number of hips	Organism profile	Success rate		Inclusion criteria
				Reported	By our criterion	
Faroug et al	2009	2	*P. aeruginosa* and MRSA (1/2) Culture-negative (1/2)	100%	100%	Patients diagnosed with chronic PJI and partial components loosen;
Anagnostakos et al.	2010	12	*S. epidermidis* and *S. aureus* (most)	91.6%	91.6%	Patients who had only late, deep septic acetabular cup loosening;
Lee et al.	2013	19	*S. aureus* and CNS (most) Culture-negative (4/19)	N.A	89.5%	Patients diagnosed with PJI and well-fixed cementless stems;
Ekpo et al.	2014	19	*S. aureus* and Streptococcus (most) MRSA (3/19) Culture-negative (4/19)	89.5%	89.5%	Patients diagnosed with chronic PJI and well-fixed stems;
Lombardi et al.	2014	7	*S. aureus* and Streptococcus (5/7) Culture-negative (2/7)	85.7%	85.7%	(1) Patients diagnosed with possible PJI and well-fixed stems; (2) Patients with a first-time infected THA in which removal of the femoral component would lead to compromise of proximal femoral bone stock; (3) Elderly patients with significant co-morbidities who had a well-fixed femoral component and poor proximal bone stock; (4) A well-fixed femoral component was directly adjacent to an ipsilateral well-fixed TKA femoral component;
Fukui et al.	2016	5	CNS (most)	80.0%	80.0%	Patients diagnosed with PJI and well-fixed stems;
Zhou et al.	2019	26	CNS (9/26) *S. aureus* (4/26) MRSA (4/26) Others (9/26)	100%	100%	Patients diagnosed with PJI and partial components loosen;
Crawford et al.	2019	41	Staphylococcus (19/41) MRSA (3/41) Others (12/41) Negative (7/41)	80.5%	80.5%	(1) Patients diagnosed with chronic PJI and well-fixed stems; (2) Removal of the femoral component would lead to significant femoral bone loss and compromise of future fixation;
Shi et al.	2020	14	Staphylococcus (12/14) *E. faecalis* (1/14) *E. coli* (1/14)	100%	92.9%	(1) Patients diagnosed with chronic PJI and partial components loosen; (2) Patients in worse health status with high risk of operation; (3) Patients with a positive culture of pre-operative aspirated synovial fluid with sensitive antibiotics.
Current study	N.A	28	Staphylococcus (18/28) MRSA (2/28) Others (2/28) Negative (6/28)	N.A	85.7%	(1) Patients diagnosed with chronic PJI and well-fixed stems; (2) Removal of the femoral component would lead to significant femoral bone loss and compromise of future fixation; (3) Elderly patients in worse health status with high risk of operation;

*MRSA* methicillin-resistant *staphylococcus aureus*, *PJI* periprosthetic joint infection, *CNS* coagulase-negative Staphylococcus, *N. A* not available, *THA* total hip arthroplasty, *TKA* total knee arthroplasty

PTE represents a promising new surgical option for the treatment of PJI; however, considerable uncertainty remains regarding whether this approach can guarantee effectiveness similar to that of TTE. To address these concerns, We retrospectively analyzed our 135 TTE patients who received TTE treatment during the same period. After adjusting for factors such as age, BMI and organism type, we found there was no significant difference in the surgical success rate between these two groups (*p* = 0.5). Lee et al. [8] retained well-fixed cementless stems as part of the treatment of infected THA for 19 patients; their overall success rate was 90%. Subsequent studies by Ekpo et al. [7] and Lombardi et al. [11] demonstrated a success rate of 89% for PTE, with an average follow-up of 4 years [7, 11]. These results were similar with our findings. Recently, another study examined the use of PTE revision surgery in 26 patients with infected THA; 100% of patients were free of infection at the time of publication and the max follow-up was approximately 6.2 years [9]. The extremely high success rate may be the result of the shorter average follow-up time in that study; however, it strongly suggests that PTE with an antibiotic-laden cement spacer is an acceptable method for the management of chronic hip PJI. We performed a retrospective statistical analysis of relative articles focusing on two-stage exchange for hip PJI. The overall success rate was 92% for 2476 TTEs in 46 studies and 89% for 57 PTEs in 5 studies [27, 28]. Although the analysis is not rigorous and there may be bias, it can still initially show that there is no significant difference between both groups in terms of success rate (*p* = 0.5).

Whether to retain the femoral stem or not is the fundamental difference between the TTE and PTE. Therefore, the key to deciding to adopt PTE lies in the judgment of whether the femoral prosthesis is infected. Unfortunately, there is no systematic analysis yet, and there are no quantifiable evaluation indicators as standards. Fukui et al. [25] proposed that it can be comprehensively determined by plain radiography, computed tomography, magnetic resonance imaging, and bone scintigraphy. For unclear cases, fuorodeoxyglucose positron-emission tomography (FDG-PET) scan can help to assess the stability of the prosthesis and determine if the infection has invaded the femur. Anagnostakos et al. [23] pointed out the antigranulocyte scintigraphy plays an important role in evaluating infection, and also mentioned that it can be further confirmed during surgery, but the exact procedure has not been clarified. Chen et al. [29] defined the criterion of femoral stem loosening as meeting any one of the following conditions: (a) subsidence > 2 mm; (b) a complete radiolucent line along the stem surface > 2 mm; (c) The endosteum became scallop-shaped; (d) Visible migration of the prosthesis. Previous studies have indicated that, if it is difficult to remove the femur implant without osteotomy, the implant can be considered stable and reliable [8, 29]. Combined with these related reports, we believe that the preoperative X-ray, intraoperative soft tissue condition around the prosthesis, and the difficulty of pulling out the femoral stem can be used to determine whether the femoral prosthesis is free of infection and is safe to retain.

Another controversial aspect regarding implementation of the PTE procedure is the interval between the two surgeries. It is generally believed that a longer period of inactivity is associated with a better outcome; however, some reports have questioned this paradigm, suggesting that a longer interval between stages may increase the chance of failure [30]. In a study of 50 patients with PJI, Haddad et al. [31] achieved a 92% infection control rate using an interval of approximately 3 weeks between surgeries. The mean time to reimplantation in most studies has been 6–12 weeks [7, 10, 11], which is slightly shorter than the mean interval of 4.3 months (range, 3–12 months) between first and second surgeries described in the present study; success rates have been broadly similar across studies. All patients in our study received oral antibiotics for at least 3 months after the second-stage surgery; this was intended to control opportunistic infections and may have improved patient outcomes.

In contrast to the approaches used in previous PTE studies, we removed the femoral head prostheses and applied additional antibiotic-loaded cement around the proximal end of the femoral stem; this approach was expected to further reduce bone loss and prevent bacteria from affecting the femoral stem components. We presume that this procedure will ultimately prove beneficial to patients, despite the lack of difference in success rates compared to other studies. Continued follow-up is needed to properly assess the long-term effects and other aspects of the procedure.

The average Harris hip score observed in this study was high at the final follow-up. This outcome may be due in part to cultural differences regarding pain tolerance in Chinese people. Within Chinese society, there is a strong sentiment that drugs should be avoided as much as possible. Accordingly, Chinese patients are often less likely to take painkillers than other groups, resulting in higher hip scores over time.

We acknowledge several limitations of this study. This was a retrospective study with a relatively small group of patients, which reduced the robustness of the conclusions, compared with a large randomized controlled trial. Furthermore, the follow-up time was relatively short, and the results may differ with a longer follow-up period. In addition, we were unable to assess clinical differences related to the virulence of the infecting organism, due to the small sample size. Nevertheless, the results of the present study provide useful insights regarding the clinical efficacy of PTE revision.

## Conclusions

PTE may be an acceptable option for a subset of patients with infected THA, offering a satisfactory infection control rate and clinical outcomes comparable to those of TTE, and with less harm. Further studies are needed to more comprehensively determine the potential applications and limits of this approach.

## Data Availability

The datasets used and/or analysed during the current study are available from the corresponding author on reasonable request.

## Abbreviations

THA: total hip arthroplasty
PTE: partial two-stage exchange
TTE: total two-stage exchange
PJI: periprosthetic joint infection
GEE: generalized estimating equations
